# Endotracheal tubes with dexamethasone eluting electrospun coating improve tissue mechanical function after upper airway injury

**DOI:** 10.1038/s41598-024-53328-1

**Published:** 2024-02-03

**Authors:** Gabriela Gonzales, Ronit Malka, Lisa Marinelli, Christine M. Lee, Solaleh Miar, Stacy Cook, Gregory R. Dion, Teja Guda

**Affiliations:** 1 Department of Biomedical Engineering and Chemical Engineering, The University of Texas at San Antonio, 1 UTSA Circle, San Antonio, TX 78249 USA; 2 Department of Otolaryngology – Head and Neck Surgery, Brooke Army Medical Center, JBSA, Fort Sam Houston, TX 78234 USA; 3 Department of Pathology and Area Laboratory Services, Brooke Army Medical Center, JBSA, Fort Sam Houston, TX 78234 USA; 4 Department of Civil, Environmental, and Biomedical Engineering, University of Hartford, West Hartford, CT 06117 USA; 5 Department of Otolaryngology – Head and Neck Surgery, University of Cincinnati College of Medicine, University of Cincinnati Medical Center, 231 Albert Sabin Way, Cincinnati, OH 45267 USA

**Keywords:** Respiratory tract diseases, Drug delivery

## Abstract

Corticosteroid-eluting endotracheal tubes (ETTs) were developed and employed in a swine laryngotracheal injury model to maintain airway patency and provide localized drug delivery to inhibit fibrotic scarring. Polycaprolactone (PCL) fibers with or without dexamethasone were electrospun onto the ETT surface PCL-only coated ETTs and placed in native airways of 18 Yorkshire swine. Regular and dexamethasone-PCL coated ETTs were placed in airways of another 18 swine injured by inner laryngeal mucosal abrasion. All groups were evaluated after 3, 7 and 14 days (n = 3/treatment/time). Larynges were bisected and localized stiffness determined by normal indentation, then sequentially matched with histological assessment. In the native airway, tissue stiffness with PCL-only ETT placement increased significantly from 3 to 7 days (*p *= 0.0016) and 3 to 14 days (*p *< 0.0001) while dexamethasone-PCL ETT placement resulted in stiffness decreasing from 7 to 14 days (*p *= 0.031). In the injured airway, localized stiffness at 14 days was significantly greater after regular ETT placement (23.1 ± 0.725 N/m) versus dexamethasone-PCL ETTs (17.10 ± 0.930 N/m, *p *< 0.0001). Dexamethasone-loaded ETTs were found to reduce laryngotracheal tissue stiffening after simulated intubation injury compared to regular ETTs, supported by a trend of reduced collagen in the basement membrane in injured swine over time. Findings suggest localized corticosteroid delivery allows for tissue stiffness control and potential use as an approach for prevention and treatment of scarring caused by intubation injury.

## Introduction

Respiratory failure is among the leading causes of hospital admissions in the United States with up to 13 million patients requiring intubation annually^1^. While intubation is a standard procedure, it is frequently associated with airway injury that can range from mucosal damage to life-threatening complications that significantly impact quality of life^2,3^. Some of the factors that increase the likelihood of developing injury include difficult intubation, incorrect tube diameter selection, high cuff pressures, or prolonged intubation^4–6^. Early complications associated with endotracheal intubation include dysphagia and dysphonia^7,8^. Intubation injury can contribute to the development of vocal process granulomas, vocal fold ulcerations, or vocal fold paralysis resulting from direct endotracheal tube contact with surrounding laryngeal tissue or compression of the recurrent laryngeal nerve from lateral pressure on the arytenoid cartilage^5^. Despite initial patient discomfort during speaking or swallowing, laryngeal evaluation generally only occurs if the symptoms persist longer than one week^2,9^. If left untreated, these injuries can result in glottic or subglottic stenosis over time leading to more severe outcomes such as airway obstruction^8,10^. Few treatment approaches exist for acute laryngeal injury.

Although treatment methods depend on the stage of injury, most approaches involve corticosteroid delivery to reduce inflammation and prevent further development of scar tissue^11,12^. However, patients generally present to clinicians long after the initial intubation, allowing acute tissue injuries to progress into mature fibrosis and requiring surgery to improve airway patency. It has been demonstrated that early interventions may reduce the need for more invasive procedures to restore laryngeal function late in injury progression^13,14^. Therefore, therapeutic strategies in the acute phase of the wound healing process could potentially reduce the severity of fibrosis, allow the retention of vocal fold function, and prevent long-term impairment.

Drug eluting endotracheal tubes (ETTs) are a potential approach for localized delivery of therapeutics to treat mucosal injury and prevent future fibrosis and scarring from a traumatic or challenging intubation. Given that both local tissue injections and systemic corticosteroids are commonly administered to manage laryngotracheal conditions^11,15,16^, a targeted steroid delivery system for localized treatment has the potential to improve drug efficacy^17^. There has been a previous report investigating the use of poly (lactic-co-glycolic) acid (PLGA) nanofibers loaded with mometasone furoate as an efficient ETT coating for reducing laryngeal mucosal thickness and submucosal laryngeal edema^18,19^. Additionally, tracheal tubes have been prepared to deliver compounds like allicin^20^, budenoside^21^, phlorotannin^22^, and lasioglossin^23^ from their external surfaces, and tested in a variety of small animal preclinical models to leverage antibacterial/anti-inflammatory properties and retain tracheal mucosal function. Although these studies highlight the benefits of targeted drug delivery platforms for the upper airway, studies that accurately simulate laryngotracheal damage acquired during prolonged intubation and demonstrate the restoration of tissue function in large physiologically relevant preclinical models over time, remain limited in the literature.

In this study, we investigated the efficacy of a drug eluting ETT for localized delivery of a commonly employed corticosteroid treatment, dexamethasone, in the setting of a simulated traumatic laryngotracheal injury model. Polycaprolactone (PCL) nanofibers loaded with dexamethasone were electropsun on the surface of commercially available ETTs. Laryngeal intubation injury was simulated in a swine injury model and ETTs with and without dexamethasone were placed for 3, 7, and 14 days (n = 3 per ETT type and timepoint). Mechanical and histological evaluation of the excised larynges were evaluated and compared to control groups both without laryngeal injury and without drug-loading ETTs to determine if localized dexamethasone delivery could inhibit fibrosis and scarring.

## Results

### Localized stiffness of the native airway

**Figure 1:**
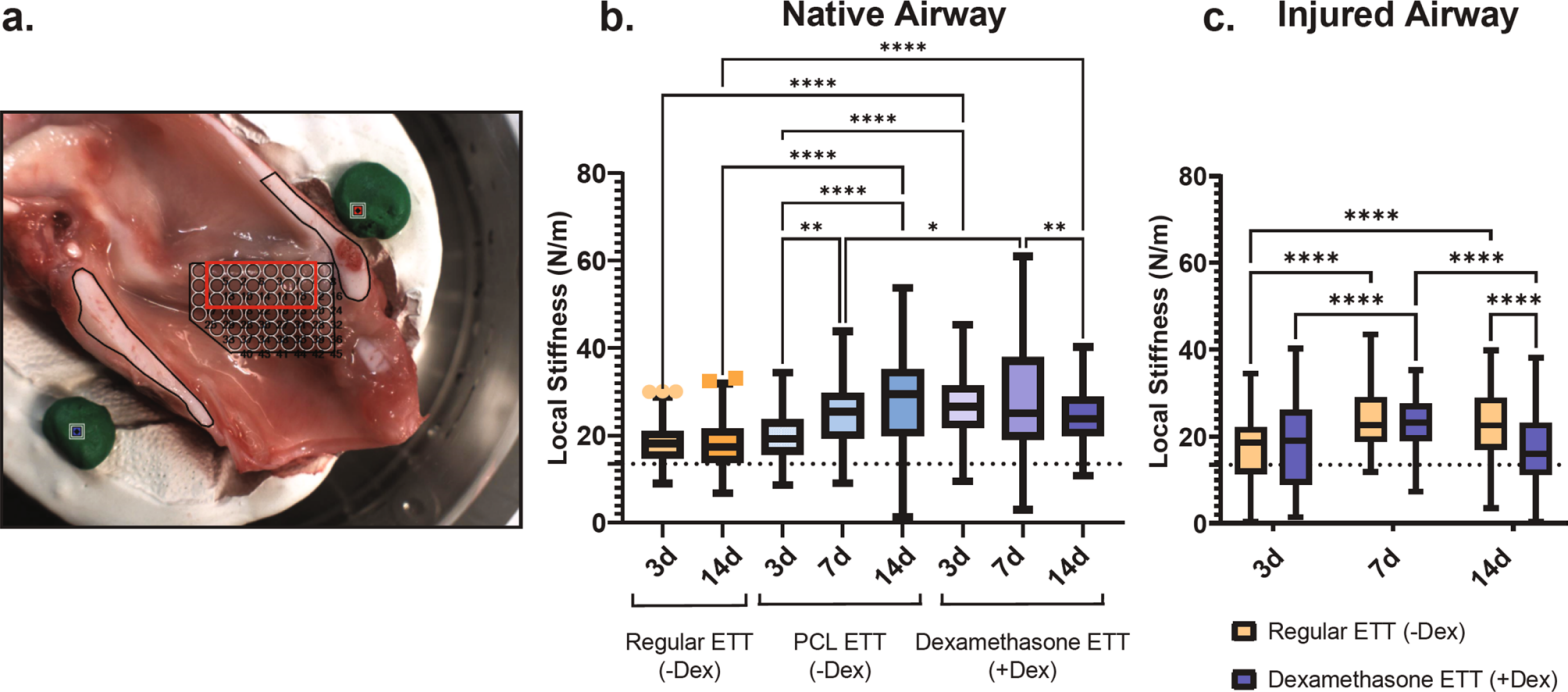
Vocal fold biomechanical outcomes. (**a**) Specimen with indentation map and inset representing region selected for analysis. Localized stiffness at 3, 7, and 14 days of (**b**) native (uninjured) airway following Regular ETT, PCL-only ETT, and dexamethasone-ETT placement and (**c**) injured airway following Regular ETT and dexamethasone ETT placement. Dashed line represents control larynges without ETT placement or injury with an estimated tissue stiffness of 13.5 N/m. (Statistically significant differences are indicated by * < .05, ** < 0.01, *** < 0.001, and **** < 0.0001).

The stiffness of the laryngeal tissue at 3 days was found to be significantly greater with dexamethasone ETT placement (26.7 ± 0.781 N/m) than regular ETTs (18.7 ± 0.534 N/m, *p *< 0.0001) and polymer-only ETTs (20.0 ± 0.524 N/m, *p *< 0.0001) (Fig. 1b). This trend remained the same for coated ETTs after 7 days with localized stiffness values significantly greater in groups with dexamethasone ETT placement (28.6 ± 1.24 N/m) than polymer-only ETT placement (24.4 ± 0.854 N/m, *p *= 0.0109). After 14 days, the groups with regular ETT placement were found to have significantly decreased stiffness outcomes (17.8 ± 0.600 N/m) than both polymer-only ETT (26.9 ± 1.10, *p *< 0.0001) and dexamethasone ETT placement (24.5 ± 0.623 N/m, *p *< 0.0001). Polymer-only ETTs resulted in a significant increase in localized stiffness from 3 days (20.0 ± 0.524 N/m) to 7 days (24.4 ± 0.854 N/m, *p *= 0.0025) and 3 days to 14 days (26.9 ± 1.10, *p *< 0.0001). On the contrary, dexamethasone ETTs resulted in a significant decrease in stiffness from 7 days (28.6 ± 1.24 N/m) to 14 days (24.5 ± 0.623 N/m, *p *= 0.0071).

### Localized stiffness of the injured airway

The stiffness at 14 days was significantly greater for tissue after placement of ETTs without dexamethasone (23.1 ± 0.725 N/m) than after the placement of ETTs with dexamethasone (17.10 ± 0.930 N/m, *p *< 0.0001) (Fig. 1c). For injured airways treated with regular ETTs, stiffness values increased significantly from 3 to 7 days and 3 to 14 days (*p *< 0.0001). The localized stiffness of tissue with dexamethasone loaded ETT placement increased significantly from 3 days (18.2 ± 1.04 N/m) to 7 days (23.6 ± 0.528 N/m, *p *< 0.0001) and decreased significantly from 7 to 14 days (17.1 ± 0.930 N/m, *p *< 0.0001).

### Histological outcomes

**Figure 2:**
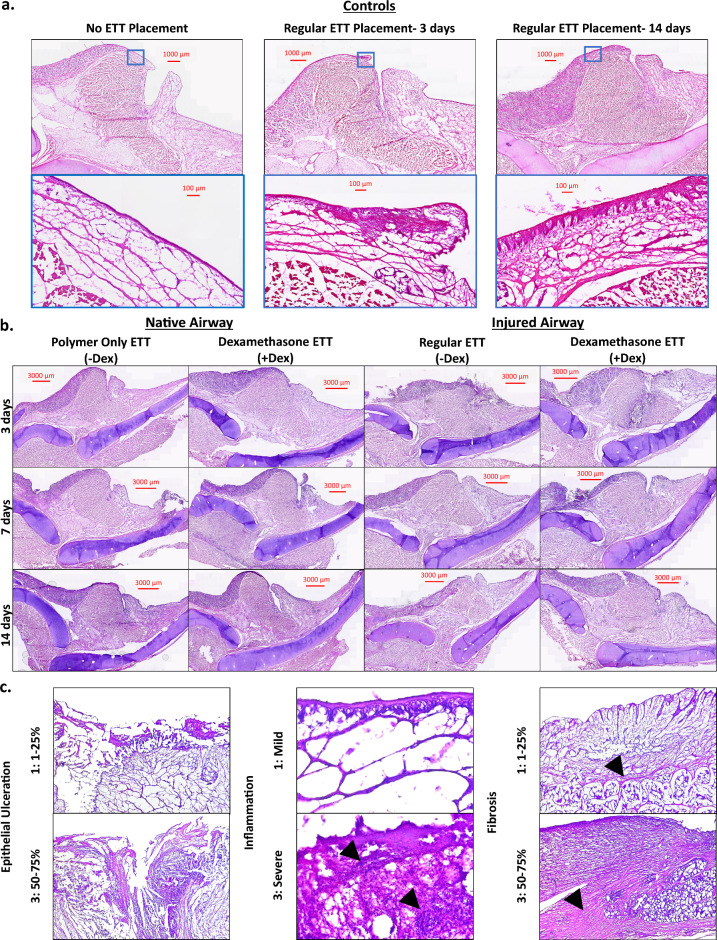
H&E-stained cross sections of (**a**) vocal fold without ETT placement or injury and clinical control with regular ETT placement and no injury after 3 and 14 days, (**b**) vocal fold in native and injured airway after ETTs with and without dexamethasone were placed for 3, 7, and 14 days, and (**c**) Higher power magnification of samples demonstrating epithelial ulceration (40x), inflammation (400x; arrowheads highlighting areas with increased inflammatory cells), and fibrosis (40x; Arrowheads highlighting collagen deposition).

Regular ETT placement in the native airway representing clinical standards demonstrated no ulceration and mild inflammation at 3 and 14 days. Polymer only ETTs in the native airway showed no ulceration but progressively increasing inflammation. In dexamethasone ETTs, there was increasing ulceration and consistent inflammation noted. A similar trend was seen in the injured laryngotracheal complex with the percentage of epithelial surface ulceration decreasing over time in both ETTs with and without dexamethasone. While inflammation in the native airway only reached severe classification at later timepoints with polymer-only ETT placement, it was severe with dexamethasone ETT placement across all time points. In injured airway groups, the severity of inflammation decreased over time. The degree of fibrosis was graded as 1 (0–4 scale; 1–25% fibrosis) across all groups and timepoints aside from dexamethasone ETT placement in the native airway at 14 days which reached a score of 2 (25–50% fibrosis). The histologic findings are summarized in Table 1 and Fig. 2**.**

**Table 1:**
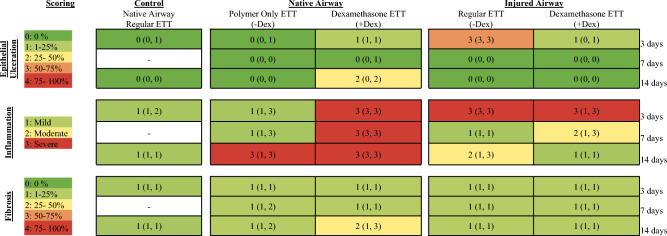
Summary of histology scoring metrics (left) and median and range scores for control, native, and injured airway with ETT placement (right).

**Figure 3:**
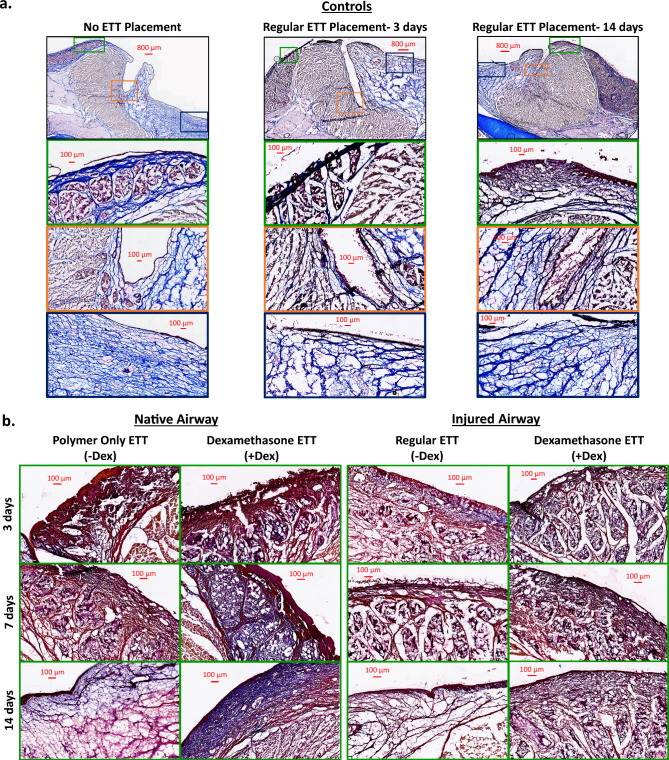
Masson’s Trichrome stained cross-sections of (**a**) vocal fold tissue without ETT placement or injury and clinical control with regular ETT placement and no injury after 3 and 14 days, (**b**) vocal fold in native and injured airway after ETTs with and without dexamethasone were placed for 3, 7, and 14 days.

**Figure 4:**
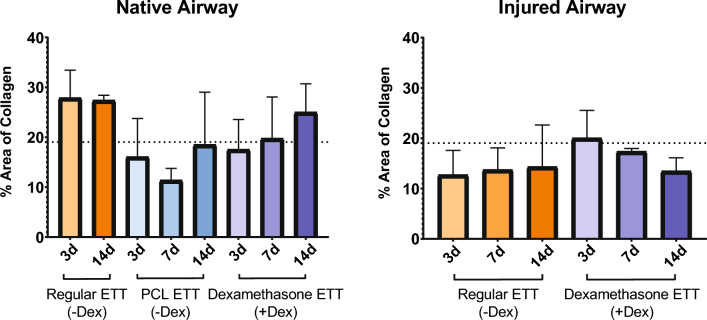
Area of collagen, expressed as percentage of the total area, determined from Masson’s Trichrome stained cross-sections of the vocal fold for the native and injured airway with ETT placement. Dashed line represents control larynges without ETT placement or injury with an estimated 19.0% area of collagen.

Masson’s Trichrome stained cross-sections and quantification are presented in Figs. 3 and 4 respectively. There were no statistical differences identified in the assessment of collagen in the laryngeal tissue with ETT placement and injury. In the native airway, there was a higher area percentage of collagen in groups with regular ETT placement at 3 and 14 days in comparison to coated ETTs and control larynges. Laryngeal tissue with dexamethasone ETT placement showed increased levels of collagen over time, while polymer only ETTs demonstrated a decrease from 3 to 7 days and increase from 7 to 14 days. In the injured airway, the area percentage of collagen was higher in groups with dexamethasone ETT placement at 3 and 7 days in comparison to regular ETT placement groups. However, after 14 days the collagen measurement remained similar in both groups.

## Discussion

In the present study, electrospun, drug eluting ETTs were developed and placed in a simulated laryngotracheal injury swine model with the goal of reducing intubation related inflammation and inhibiting the development of vocal fold scarring. Our objective was to determine differences in the mechanical properties of laryngeal tissue with ETT placement after simulated intubation injury and use mechanical stiffness as a quantifiable metric for tissue function and/or initiation of scarring leveraging a previously developed approach^24^. Vocal fold biomechanics have previously been characterized by the cover-body theory where the epithelium and superficial layer of the lamina propria represents the cover surface and the underlying thyroarytenoid (TA) muscle corresponds to the body^25^. Injury to this mucosal interface, such as with endotracheal intubation in the setting of vocal fold injury, can lead to dysfunction in the vibratory behavior required for modulating airflow and phonation^26^. Ultimately, this may lead to scar tissue formation that further impedes function as the pliability of the vocal folds is reduced and stiffness increases^27,28^. Investigations of vocal fold scarring in injured animal models^29,30^ have found these tissue property changes to be correlated with the organization of collagen and elastin bundles during the wound healing process^31,32^. Despite the considerable insight of the vocal fold wound healing process, an effective treatment for scarring remains a clinical challenge.

Localized drug delivery to the injured laryngeal mucosa can be effectively achieved from coated, drug-eluting devices such as ETTs^18,19,33–36^. One implementation of this technology was used to reduce the incidence of ventilator-associated pneumonia by utilizing silver-coated ETTs to prevent biofilm formation and bacterial colonization^37^. Another approach was focused on modulating upper-airway bacterial infection during intubation by incorporating an antimicrobial peptide coating on the surface to target microorganisms^36^. While findings suggested that coated ETTs reduced the incidence of pathogens associated with subglottic stenosis and did not permit any bacterial adherence to the tube, these ETTs were primarily intended to eliminate pathogenic bacteria and not for a targeted anti-inflammatory effect. Other studies exploring the use of a mometasone furoate eluting ETT in the laryngotracheal lumen of rats showed promising findings for reducing mucosal thickness and fibrosis in comparison to uncoated ETTs^18,19^. In that formulation, the electrospun PLGA nanofibers were used to control the release of the mometasone furoate, and the system was tested in vivo in a rat tracheal model initially for 24 hours^18^ to assess submandibular hypertrophy, and in a second study for 1 week^19^ where picrosirius red staining was used to determine collagen content in the mucosa. Interestingly, after 1 week in rats, the PLGA fibers alone (no drug) increased collagen content significantly compared to the control groups or those with incorporated steroid delivery which were both similar, much like we observed in the present study (Fig. 4). PLGA has been used to deliver a variety of compounds (analgesics^35^, anti-bacterials^38^ and anti-inflammatories) form the surface of tracheal grafts, tracheal tubes and ETTs, both in the form of fibers^19^ as well as solid film coatings^35,38^. The difference between films and fibers in coating is the degree of control (dose and kinetics) in the release of the incorporated drug locally. Similarly, we use PCL for our electrospun coating because it is a slower degrading material compared to PLGA. There is a previous report of PCL used for dip-coating ETTs, and loaded with phlorotannin to prevent tracheal stenosis, which was tested in a rabbit tracheal injury for 1 week placement and 4 weeks extended followup^22^, showing similar results of reduced collagen production compared to uncoated ETTs. A different study developed an electrospun PCL melt-writing process to generate a tracheal scaffold^39^, which was then loaded with dexamethasone coating and used to restore a tracheal segmental defect also in a rabbit model. While that study was primarily focused on looking at pro-regenerative compared to anti-fibrotic behavior in the graft, they show PCL is well tolerated in this environment and use qualitative histological assessment with Masson’s trichrome stain similar to our approach. The primary outcomes in our study were focused on evaluating the longitudinal maintenance or recovery of mechanical tissue properties, specifically in a model where the tube was placed across the laryngotracheal complex throughout the study. To our knowledge, this is the first study to examine this drug delivery platform in a large animal model that is more accurately representative of the size/anatomy of the human larynx.

When observing the native airway groups (without injury), the dexamethasone ETT demonstrated higher tissue stiffness than with polymer-only ETT and regular ETT placement at early timepoints. While the tissue stiffness values increased over time with polymer-only ETT placement, it decreased with dexamethasone ETT placement. However, localized stiffness seemed to remain the same in groups with regular ETT placement after 3 and 14 days. The histologic assessment indicated higher scores for epithelial ulceration and inflammation in dexamethasone groups than polymer and regular ETT groups while minimal changes in fibrosis were identified. The collagen production in the laryngeal tissue was increased in groups with regular ETT placement and decreased in groups with polymer only ETT placement. The groups with dexamethasone ETT placement yielded an increasing percentage area of collagen over time with greater levels identified at 14 days in comparison to control laryngeal tissue.

In the laryngotracheal injured groups, we observed a similar pattern of the dexamethasone ETTs causing an initial increase in stiffness and subsiding over time. By contrast, regular ETT placement had an increasing trend with stiffness values greater than those with dexamethasone ETT placement at the latest timepoint. Inflammation scores were greatest at 3 days and decreased over time, especially in groups with dexamethasone delivery. While uninjured groups with regular ETT placement had increased percentage area of collagen, injured groups with the same treatment had decreased collagen with minimal changes over time. The dexamethasone ETTs demonstrated inverse trends to uninjured groups for collagen production with decreasing levels over time. These findings suggest that the sustained release of dexamethasone can provide longer term anti-inflammatory action, however, affects the production of collagen during the acute wound healing phase.

The primary goal in the management of laryngotracheal injuries is the restoration of the biomechanical function and maintaining a patent airway. To achieve this goal, an understanding of the tissue properties is critical for targeting treatments that restore native, or near native, function. Our group has previously investigated the mechanical properties of the larynx using normal indentation across different species and injury models ^24,40–42^. We have found this technique effective and reproducible for the quantification of the biomechanical changes associated with vocal fold injury and therapeutic interventions. Interestingly, the type of airway injury seems to have a different effect on the mechanical properties of the vocal fold. While we investigated the native airway without injury, the tissue stiffness values for non-injured and injured groups were within a similar range, suggesting intubation in and of itself increases vocal fold stiffness. Given the presence of the endotracheal tube that moves during patient motion and vocal fold motion in the non-paralyzed and less sedated patient, these stiffness increases match clinical suspicions of the negative impact of intubation on vocal fold vibratory function. However, as anticipated, dexamethasone seems to modulate epithelial mucosal inflammation over time and supports the potential role of continuous release from an endotracheal tube. Further investigation is necessary to determine how these outcomes are correlated to the vibratory function of the vocal fold and subsequent phonation.

There were limitations to our study that merit consideration. Due to our abrasion method for de-epithelialization, the airway injuries varied across samples leading to differences in the severity of vocal fold scarring over time. Areas of injury were preferentially identified on gross evaluation and from endoscopic images for comparison, though subtle differences in injury depth and breadth may contribute to results variability within and across groups. Given the injured airway model injuries were superficial, we selected a smaller indenter tip and indentation depth for mechanical analysis. However, alternative selections may have provided additional tissue properties beyond the superficial layer. Our experiment was primarily focused on mechanical outcomes to quantify tissue properties, but after conducting our biomechanical tests the larynges were refrozen before sectioning for histologic evaluation. The resultant freezing artifact may have limited recognition of more subtle histologic alterations such as early collagen deposition.

## Conclusion

The type of ETT, with or without corticosteroids, and the duration of intubation contribute to changes in vocal fold biomechanical properties in both native and injured airways. For this injury model simulating intubation injury, corticosteroid delivery from the surface of dexamethasone-PCL coated ETTs reduced vocal fold stiffness over 14 days of intubation. These findings suggest localized delivery of dexamethasone via ETT electrospun fiber coating allows for the control of tissue stiffening and can potentially be used as an approach for prevention and treatment of laryngeal scarring in patients requiring long-term intubation.

## Materials and methods

### Animals

All procedures contributing to this work comply with the ethical standards of the BRIDGE PTS Institutional Animal Care and Use Committee (Protocol BPTS-21-01). Methods were carried out in accordance with approved guidelines and regulations described by the Guide for the Care and Use of Laboratory Animals. This study was reported as recommended by the Animal Research: Reporting of In Vivo Experiments (ARRIVE) guidelines.

### Endotracheal tube preparation

Dexamethasone loaded polycaprolactone (PCL) fibers were electrospun on the surface of commercially available endotracheal tubes (ETTs). Briefly, PCL (Mw = 80,000) was dissolved in chloroform (15:85 w/w). Dexamethasone sodium phosphate was added to the homogeneous mixture at a concentration of 10% (w/w) of the total polymer mass along with ethanol used as its solvent. For polymer-only ETTs, dexamethasone was not added to the blend. The solution was loaded into a Luer Lock syringe and dispensed from a blunt needle using a pump (Pump11 Elite, Harvard Apparatus, Holliston, MA) at an infusion rate of 1.8 mL/hr. A 5 cm section of a 7.0 mm inner diameter ETT (Shiley™ Lo-Pro Oral/Nasal Tracheal Tube Cuffed, Covidien, Mansfield, MA) was positioned on a rotating (300 rpm) collector 20 cm below the needle tip where a voltage of 20 kV was applied (Gamma High Voltage Research, Ormond Beach, FL). The decision to use only a portion of the ETT was made to allow for this to act as a stent and permit the animals to maintain normal activity during the study duration in lieu of remaining sedated and intubated for prolonged periods. All chemicals were purchased from Sigma-Aldrich (St. Louis, MO).

### ETT characterization

**Figure 5:**
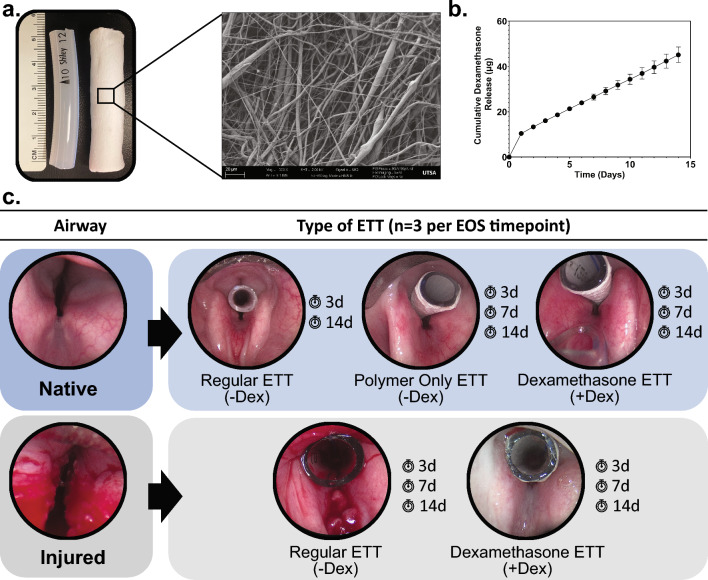
ETT characterization and schematic diagram of the experimental design (**a**) 5 cm segment of regular ETT and dexamethasone coated ETT with SEM inset exhibiting fibers (**b**) Cumulative drug release of dexamethasone from ETT spanning 14 days and (**c**) Endoscopic images of the airway with and without ETT placement. Regular, polymer only, and dexamethasone loaded ETTs were placed in the native (uninjured) airway. Regular and dexamethasone loaded ETTs were placed in an injured airway for 3, 7, and 14 days.

Scanning electron microscopy (SEM) was used to evaluate the morphology of the electrospun fiber coated ETT (Fig. 5a). Samples were sputter-coated with silver-palladium (Cressington Scientific Instruments, Watford, UK) and imaged under 2 kV applied voltage at 500X magnification using a Zeiss Crossbeam 340 Focused Ion Beam (FIB)-SEM (ZEISS, Oberkochen, Germany). Drug release from the ETT coating was assessed over 14 days (Fig. 5b). Briefly, ETT samples (n = 5) were maintained in PBS buffer at 37 °C and dexamethasone concentration was determined by measuring the absorbance of the drug in the release medium using a plate reader (Synergy2, BioTek, Winooski, VT) at a wavelength of 290 nm. PBS was replaced with fresh PBS each day.

### Injury simulation

**Figure 6:**
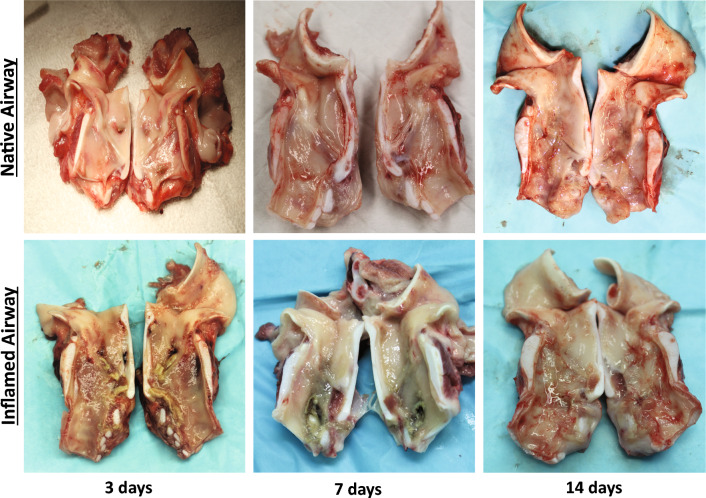
Bisected larynges with and without injury at 3, 7, and 14 days.

Thirty-six Yorkshire crossbreed swine were used to model prolonged intubation with or without laryngotracheal injury. Animals were premedicated with atropine (0.05 mg/kg), anesthetized via intramuscular injection of tiletamine-zolazepam (4.0- 8.0 mg/kg), and maintained using isoflurane during injury and endotracheal tube (ETT) placement. For groups with laryngeal injury, trauma was simulated under endoscopic visualization by mechanically abrading the airway mucosa with a 3/8 in stainless steel tube brush. The injury extent was confirmed endoscopically. A 5 cm section of either a regular ETT or a dexamethasone coated ETT were placed within the injured airway. For material control, groups without injury had either a polymer only coated ETT or a dexamethasone coated ETT for placement. The ETTs were secured transcervically with a 2–0 prolene suture and a surgical button positioned at the midline of the neck for 3, 7, or 14 days (n = 3 per each group and time point). Regular ETTs were also placed in the uninjured airway at early and late timepoints (3 and 14 days, n = 3) to allow comparison of coated ETTs to clinical outcomes. In addition, larynges without ETT placement or injury (n = 4) were obtained via tissue sharing to obtain a baseline for biomechanical properties of healthy tissue. Figure 5c shows a schematic of the experimental design. Following end of study time points, swine were euthanized using sodium pentobarbital (110 mg/kg), larynges were excised, bisected in the sagittal plane (Fig. 6), and frozen at − 80 °C until further analysis.

### Normal indentation

The larynges were thawed at 4 °C and fixed into a Plaster of Paris sample holder as previously described^40^. A Biomomentum Mach-1 v500css (Laval, Quebec, Canada) mechanical tester with a 1.5 N uniaxial load cell was used to perform normal indentation along the vocal fold region of the bisected larynges (Fig. 1a) submerged in phosphate buffered saline (PBS) to maintain moisture. A 0.3 mm amplitude test was performed at a velocity of 1.2 mm/s using a 2 mm spherical indenter tip. The tissue stiffness was determined from the normal indentation force versus displacement graphs.

### Histology and scoring

Following mechanical analysis, 5 mm thick tissue sections were taken from the mid-section of the vocal fold and fixed in 4% formalin for histological evaluation. After 24 h, tissue was mounted in embedding molds with optimal cutting temperature compound (Scigen Tissue Plus O.C.T. Compound, Thermo Fisher Scientific, Waltham, MA) and stored at − 80 °C. The frozen specimens were sectioned using a Cryostat (Eredia™ NX70, Kalamazoo, MI) and affixed onto glass slides. Samples were stained with hematoxylin and eosin (H&E) and Masson’s Trichrome according to the manufacturers protocol (Newcomer Supply, Middleton, WI). A Motic EasyScan Pro 6 Slide Scanner (Motic Instruments, Schertz, TX) was used to image the slides at 20X. H&E stained slides were independently reviewed by two pathologists for the assessment of epithelial ulceration, inflammation, and fibrosis. Scores with differing assessments of more than one point were re-reviewed by both pathologists to achieve consensus. Epithelial ulceration and fibrosis were graded on a five-point scale based on the percentage of surface epithelium which was ulcerated or percentage of the submucosa inferior to the vocalis muscle occupied by fibrosis, respectively (0: 0%, 1: 1–25%, 2: 25–50%, 3: 50–75%, 4: 75–100%). The degree of inflammation was graded on a three-point scale (1: mild, 2: moderate, 3: severe). The area of collagen, expressed as percentage of the total area, was measured in ImageJ (version 1.53 k, National Institute of Health, USA) using the color deconvolution2 plugin^43^. The imported image was decomposed into 3 colors corresponding to Masson’s Trichrome stain using the built-in vectors with the blue component representative of collagen. The threshold was adjusted to 0–105, and the percentage of pixels highlighted red in the laryngeal tissue above the thyroid and cricoid cartilage was determined using the measure tool.

### Statistical analysis

Mechanical properties are reported as mean ± standard error and histology scores were reported as median (range). For the evaluation of localized stiffness and area of collagen, analysis of variance (ANOVA) was performed across the variables (ETT type and time) followed by Tukey’s multiple comparisons test to identify significant differences (*p *< 0.05). Statistical analysis was conducted with GraphPad Prism (v9.4.0 for Windows, San Diego, California).

### Supplementary Information

Supplementary Information.

## Data Availability

All data generated during and/or analyzed during the current study are available in Supplementary information files.
